# Dermatomyositis and Polymyositis in the Intensive Care Unit: A Single-Center Retrospective Cohort Study of 102 Patients

**DOI:** 10.1371/journal.pone.0154441

**Published:** 2016-04-26

**Authors:** Jin-Min Peng, Bin Du, Qian Wang, Li Weng, Xiao-Yun Hu, Chan-Yuan Wu, Yan Shi

**Affiliations:** 1 Department of Medical Intensive Care Unit, Peking Union Medical College Hospital, Peking Union Medical College & Chinese Academy of Medical Sciences, Beijing, China; 2 Department of Rheumatology, Peking Union Medical College Hospital, Peking Union Medical College & Chinese Academy of Medical Sciences, Beijing, China; 3 Department of General Intensive Care Unit, Peking Union Medical College Hospital, Peking Union Medical College & Chinese Academy of Medical Sciences, Beijing, China; National Institutes of Health, UNITED STATES

## Abstract

**Introduction:**

Patients with idiopathic inflammatory myopathies (IIMs) are sometimes complicated with life-threatening conditions requiring intensive care unit (ICU) admission. In the past, owing to the low incidence of IIM, little was known about such patients. Our aim was to investigate the clinical features and outcomes of these patients and identify their risk factors for mortality.

**Methods:**

A retrospective study was performed of IIM patients admitted over an 8-year period to the medical ICU of a tertiary referral center in China. We collected data regarding demographic features, IIM-related clinical characteristics, reasons for admission, organ dysfunction, and outcomes. Independent predictors of ICU mortality were identified through multivariate logistic regression analysis.

**Results:**

Of the 102 patients in our cohort, polymyositis (PM), dermatomyositis (DM), and clinically amyopathic dermatomyositis (CADM) accounted for 23.5%, 64.7%, and 11.7% respectively. The median duration from the onset of IIM to ICU admission was 4.3 months (interquartile range [IQR], 2.6–9.4 months). Reasons for ICU admission were infection alone (39.2%), acute exacerbation of IIM alone (27.5%), the coexistence of both (27.5%), or other reasons (5.8%). Pneumonia accounted for 97% of the infections; 63.2% of infections with documented pathogens were caused by opportunistic agents. Rapid progressive interstitial lung disease (RP-ILD) was responsible for 87.5% of acute exacerbation of IIM. The median Acute Physiology and Chronic Health Evaluation II (APACHE II) score on ICU day 1 was 17 (IQR 14–20). On ICU admission, acute respiratory failure (ARF) was the most common type (80.4%) of organ failure. The mortality rate in the ICU was 79.4%. Factors associated with increased ICU mortality included a diagnosis of DM (including CADM), a high APACHE II score, the presence of ARF, a decreased PaO_2_/FiO_2_ ratio, and a low lymphocyte count at the time of ICU admission.

**Conclusions:**

The outcome of IIM patients admitted to the ICU was extremely poor. A diagnosis of DM/CADM, the presence and severity of ARF, and the lymphocyte counts at ICU admission were shown to be valuable for predicting outcome. Opportunistic infections and rapidly progressive interstitial lung disease warrant concern in treating these patients.

## Introduction

Idiopathic inflammatory myopathies (IIMs) are a group of systemic rheumatic diseases (SRDs) of unknown etiology that mainly involve the skeletal muscles. These diseases are characterized by muscle weakness and elevated muscle enzymes in serum. Polymyositis (PM) and dermatomyositis (DM) are two typical subtypes of IIM, whereas clinically amyopathic dermatomyositis (CADM) is a newly recognized subgroup of DM wherein only the typical skin rash of DM appears, without any evidence of muscular impairment [1, 2]. Patients with IIMs represented only about 4.5% to 11.3% of SRDs in the ICU [3–6], their prognoses were poor. Fatal respiratory and cardiac complications included rapid progressive interstitial lung disease (RP-ILD), respiratory muscle weakness, and heart failure [7, 8] as well as severe infection [9, 10]. Moreover, the medical complexity of IIMs poses a considerable challenge to the intensivists owing to the difficulty of arriving at a differential diagnosis and the therapeutic dilemmas posed by acute exacerbations of IIM and severe infection.

So far approximately 70 IIM patients have been included in the reports on SRD patients admitted to an ICU [3, 5, 6, 11–15]. However, only one study, by Sherer and colleagues, focuses exclusively on IIM patients. It describes 6 DM patients consecutively admitted in the ICU over a 12-year period, but because of the small sample size does not offer enough detail to permit identification of the prognostic factors [15]. We therefore decided to conduct a large retrospective cohort study in order to delineate clinical features and outcomes and to investigate the risk factors for mortality in this group of patients.

## Materials and Methods

### Patients

We performed an electronic search using the ICD10 codes “polymyositis” and “dermatomyositis” to identify patients with a diagnosis of PM/DM admitted to the medical ICU in Peking Union Medical College Hospital (PUMCH) between January 1, 2006, and January 1, 2014. PUMCH is one of several nationwide referral centers in China for patients with rheumatic disease. The medical ICU at PUMCH had 15 beds and admitted about 100 to 110 SRD patients annually. All patients with PM/DM fulfilled the definite or probable diagnostic criteria of Bohan and Peter [16]; the diagnosis of CADM was based on the criteria developed by Sontheimer and colleagues [17]. Juvenile PM/DM and IIM secondary to malignancies, infections, or other SRDs were excluded.

This study was approved by the Institutional Review Board of Peking Union Medical College Hospital (PUMCH) and the Chinese Academy of Medical Science (CAMS). Patient approval or informed consent was waived because the study involved a retrospective review of patient records.

### Data collection

The ICU’s internal database was searched and patients’ medical charts were retrospectively reviewed. Where patients were readmitted, the second ICU stay was not included in this study. For all patients the following data were collected: (1) pre-ICU information such as demographics, disease course of IIMs, IIMs-related clinical manifestations and laboratory findings, major organ involvement and immunosuppressive therapy within 1month prior to ICU admission; 2) ICU-related data, such as primary causes of ICU admission, major laboratory findings including biochemical parameters at ICU admission and microbiological results, severity-of-illness score (Acute physiology and Chronic Health Evaluation II (APACHE II) score and Sequential Organ Failure Assessment (SOFA) score on the first ICU-day, complications, supportive treatment, and outcomes.

### Definition

#### Causes for ICU admission

The primary reasons for ICU admission were classified into 4 groups: (1) acute exacerbation of IIM, defined as non-infectious manifestations directly attributable to the acute flare of IIMs—for instance, RP-ILD, respiratory muscle weakness or congestive heart failure; (2) infections, defined as microbiologically documented or clinically presumed infections, such as pneumonia or septic shock; (3) concomitant infection and exacerbation of IIM; (4) others including postoperative care and acute serious illness unrelated to IIM.

All patients were consulted by rheumatologists during their ICU stay. Medical records were reviewed and the reasons for admission were confirmed by the consensus of 2 intensivists (JMP and LW) and 2 rheumatologists (QW and CYW) according to clinical data and therapeutic response.

#### ICU-related conditions

Shock was defined as systolic blood pressure <90 mmHg despite adequate fluid resuscitation or need for vasopressors for at least 1 hour. Acute respiratory failure (ARF) was defined as the ratio of a partial pressure of oxygen in arterial blood to a fraction of inspired oxygen (PaO_2_/FiO_2_) <200 mmHg with mechanical ventilation. Acute kidney injury (AKI) was diagnosed according to Kidney Disease Improving Global Outcomes criteria [18].

#### Definition of infections

Diagnosis of pneumonia was based on clinical, radiographic, and microbiological findings. Bacterial pneumonia was defined as the presence of 10^4^ colony-forming units per milliliter in bronchoalveolar lavage fluid. Invasive pulmonary aspergillosis in this study included proven and probable invasive aspergillosis, diagnosed according to the consensus definitions of the 2008 European Organization of the Research and Treatment of Cancer/ Mycoses Study Group [19]. *Pneumocystis jiroveci* pneumonia was diagnosed according to the positive silver staining yield from respiratory samples or a positive polymerase chain reaction test for *P*. *jiroveci* DNA with classic radiological findings and an appropriate response to the standard treatments for *P*. *jiroveci* pneumonia [20]. Cytomegalovirus (CMV) pneumonia is diagnosed by a combination of classic imaging findings and the detection of CMV in bronchoalveolar lavage fluid or lung tissue samples [21].

#### IIMs-related conditions

ILD associated with IIMs (IIMs-ILD) was diagnosed based on typical clinical manifestations and consistent abnormalities observed in high-resolution CT scans of the lungs. Notably, most patients with IIMs-ILD were critically ill and hence unable to undergo pulmonary function tests. RP-ILD was defined as the development or worsening of dyspnea without evidence of overt infection within 1 month that required high-flow oxygen therapy or ventilator support in previously asymptomatic patients or ones with mild shortness of breath. High-dose glucocorticoid therapy was defined as doses equivalent to more than 0.5 mg/kg daily of prednisone.

### Statistical analysis

The normality of continuous variables was tested by the Kolmogorov-Smirnov goodness-of-fit model. Continuous variables were reported as mean ± SD if normally distributed and as median within the interquartile range (IQR) if skewed. Categorical variables were reported as frequencies and percentages. Parameters were compared between survivors and non-survivors first by univariate analyses. The Fisher exact test or chi-square tests were conducted in order to compare binary and categorical variables. Independent sample 2-tailed *t*-tests or the Mann-Whitney test was used for continuous variables. Factors with *P* values <0.1 in the univariate analyses were investigated further in multivariate logistic regression analysis (stepwise backward entry) to determine the independent predictors of ICU mortality. We used 2 separate models: one for demographics, conditions related to IIMs, ICU admission status, severity of illness, organ failures at ICU admission, and interventions and the other replacing parameters about organ failures and interventions with laboratory findings at ICU admission. Variables were tested for linearity and interactions before inclusion in the multivariate models. Goodness of fit of the model was evaluated by the Hosmer-Lemeshow statistic. The Kaplan-Meier survival curve was used to depict the 28-day ICU mortality of patients with PM and DM/CADM. The log-rank statistic was used to test the difference. *P* values <0.05 were considered statistically significant. The statistical analyses were performed with SPSS statistics software (version 20.0; SPSS Inc., Chicago, IL).

## Results

From January 2006 through January 2014, a total of 109 patients with IIMs were admitted to the ICU, including 24 with PM, 66 with DM, 12 with CADM, and 7 with malignancy-related DM. After excluding the latter, 102 patients were included in the study.

### Clinical characteristics of idiopathic inflammatory myopathy

The clinical characteristics of IIM are summarized in Table 1. Forty-five (44.1%) patients were male; their mean age was 50 years. The median duration from the onset of IIMs to ICU admission was 4.3 months. IIMs-ILD was complicated in 79 (77.5%) patients; it was more prevalent in patients with DM/CADM than in those with PM (84.6% vs. 54.2%, *P* = 0.002). Anti-nuclear antibody and anti-Jo-1 antibody were identified in 23 and 2 patients respectively. Within 1 month prior to ICU admission, 71 patients received high-dose glucocorticoids and 67 received immunosuppresants. In addition, owing to acute life-threatening exacerbation of IIM, 26 patients and 30 patients received methylprednisolone pulse therapy and intravenous immunoglobulin (IVIG) respectively. Protean immunosuppressants included cyclophosphamide (n = 44), followed by methotrexate (n = 20), cyclosporine A (n = 8), hydroxychloroquine(n = 3), mycophenolate mofetil (n = 2), tacrolimus (n = 2), and azathioprine (n = 1). Because of the strong concern of infection, no patients received additional immunosuppressants during their ICU stay.

**Table 1:** Clinical characteristics of the 102 patients with Idiopathic Inflammatory Myopathy admitted to the ICU.

Variables	Total	Survivors	Nonsurvivors	*P* values
	(n = 102)	(n = 21)	(n = 81)	
Male, n (%)	45 (44.1)	5 (23.8)	40 (49.4)	0.035
Age, mean (SD)	50.5 (13.7)	50.9 (14.3)	50.4 (13.6)	0.880
Duration of IIM before ICU admission (month), median (IQR)	4.3 (2.6, 9.4)	4.0 (1.5, 10.9)	4.3 (2.7, 8.7)	0.484
**IIM types, n (%)**				
PM	24 (23.5)	12 (57.1)	12 (14.8)	< 0.001
DM	66 (64.7)	8 (38.1)	58 (71.6)	0.004
CADM	12 (11.8)	1 (4.8)	11 (13.6)	0.461
**Comorbidity, n (%)**				
Diabetes mellitus	10 (9.8)	0 (0)	10 (12.3)	0.199
Coronary heart disease	9 (8.8)	3 (14.3)	6 (7.4)	0.386
Hypertension	17 (16.7)	3 (14.3)	14 (17.3)	1.000
**Systemic involvement related to IIM, n (%)**				
Raynaud’s phenomenon	5 (4.9)	1 (4.8)	4 (4.9)	1.000
Vasculitis	18 (17.6)	3 (14.3)	15 (18.5)	0.895
Arthritis/arthralgia	41 (40.2)	8 (38.1)	33 (40.7)	0.611
Dysphagia	21(20.6)	4 (19.0)	17 (21.0)	1.000
Interstitial lung disease	79 (74.5)	11 (52.4)	68 (83.9)	0.005
Spontaneous pneumomediastinum/pneumothorax	28 (27.5)	4 (19.0)	24 (29.6)	0.333
Heart	7 (6.9)	3 (14.3)	4 (4.9)	0.305
Respiratory muscle	5 (4.9)	3 (14.3)	2 (2.5)	0.095
**Recent specific therapy for IIMs**,^a^ **n (%)**				
Immunosuppressant	67 (65.7)	12 (57.1)	55 (67.9)	0.355
Methylprednisolone pulse	40 (39.2)	4 (19.0)	36 (44.4)	0.034
IVIG	45 (44.1)	6 (28.6)	39 (48.1)	0.107
High-dose corticosteroid ^b^	88 (86.3)	16 (76.2)	72 (88.9)	0.132

^a^ Therapy received within 1 month prior to ICU admission or during the first month of ICU stay.

^b^ Defined as the dose equivalent to more than 0.5 mg/kg daily of prednisone.

CADM, clinically amyopathic dermatomyositis; DM, dermatomyositis; ICU, intensive care unit; IIM, idiopathic inflammatory myopathy; IQR, interquartile range; IVIG, intravenous immunoglobulin; PM, polymyositis; SD, standard deviation.

### Reasons for ICU admission

Approximately half of the patients (49%) were directly admitted from the emergency room. Forty-eight patients were transferred from the ward; the median time from hospitalization to ICU admission was 7.0 days (IQR 2.0–18.5). Of these patients, 14 stayed in the ward for less than 48 hours before ICU admission. Infection (n = 40, 39.2%) was the leading cause of ICU admission, followed by acute exacerbation of underlying IIMs (n = 28, 27.5%) and coexistence of both (n = 28, 27.5%). Rare causes included postoperative care (n = 4), hypertensive crisis (n = 1), and severe hyponatremia (n = 1).

The sites of infection and identified pathogens at the time of ICU admission are presented in Table 2. In 68 patients with infection, the lung was the most commonly infected site. In addition multi-site infections were found in 8 patients. Causative pathogens were identified in 43 (63.2%), most being opportunistic infections (n = 30). The 3 most common of these were invasive pulmonary aspergillosis (n = 18), *P jiroveci* pneumonia (n = 11), and CMV pneumonia (n = 6). Moreover, polymicrobial infections were documented in 15 (22.1%) patients. Fifty-three (80.3%) patients had pneumonia were superimposed on underlying ILD, with a mortality of 88.7%. There was no statistically significant difference in the rate of opportunistic infection between PM patients and DM/CADM patients. In term of antibiotics use, 75% (32 of 43) of patients received proper antibiotics within 48 hours of ICU admission, while 8 of 11 patients who did not receive proper antibiotics in time were infected with opportunistic pathogens.

**Table 2:** The sites and causative organisms of infection in patients with Idiopathic Inflammatory Myopathy admitted to the ICU.

Variables	Values
**Number of patients with infection, n**	**68**
**Site of infection** ^a^, **n (%)**	76 (100)
Lung	66 (86.8)
Central nervous system	4 (5.3)
Bloodstream	3 (3.9)
Skin and soft tissue	2 (2.7)
Abdomen/Gastrointestinal tract	1 (1.3)
**Microbiological documentation** ^b^	59(100)
**Gram-negative bacteria**	14 (23.7)
*Pseudomonas aeruginosa*	2 (3.4)
*Acinetobacter baumannii*	8 (13.5)
*Escherichia coli*	2 (3.4)
*Klebsiella spp*	1 (1.7)
*Enterobacter cloacae*	1 (1.7)
**Gram-positive bacteria**	9 (15.3)
*Staphylococcus aureus*	4 (6.8)
*Enterococcus faecium*	2(3.4)
*Streptococcus pneumoniae*	1(1.7)
*Listeria monocytogenes*	1 (1.7)
*Nocardia*	1 (1.7)
**Fungi**	29 (49.2)
Invasive pulmonary aspergillosis	18 (30.5)
*Pneumocystis jirovecii*	11 (18.7)
**Viruses**	7 (11.9)
Cytomegalovirus	6 (10.2)
Influenzae	1 (1.7)

^a^ Sum of infection site exceeds 68 patients because of 8 patients with multisite infections.

^b^ Among the 68 patients with infection, 43 patients harbored documented microbial agents. The sum of pathogens isolated exceeds 43 because 15 patients had polymicrobial infections.

Exacerbation of IIMs accounted for the ICU admission of 56 patients. Among them, 49 (87.5%) presented with RP-ILD, 3 patients had hypercapnic respiratory failure due to severe respiratory muscle weakness, 2 experienced malignant arrhythmia or cardiogenic shock due to severe IIM-induced cardiomyopathy, and the other 2 had AKI due to rhambdomyolysis.

### Complications and interventions in the ICU

Table 3 describes patients’ complications and management at ICU admission and during their ICU stays. Median APACHE II and SOFA scores on ICU day 1 were 17 and 7 respectively. ARF was the predominant type of organ failure (n = 82, 80.4%), with a median PaO_2_/FiO_2_ ratio of 84 mmHg. The causes of ARF were RP-ILD (n = 19, 23.2%), pneumonia (n = 36, 43.9%), and the combination of both (n = 27, 32.9%). Septic shock was diagnosed in 46 (45.1%) patients at ICU admission; another 5 developed this condition during their ICU stay. AKI occurred in 19 (18.6%) patients. Eleven patients had ICU-acquired infections, 6 of which were bloodstream infections.

**Table 3:** Complications and intervention at ICU admission or during the ICU stay.

Variables	Total	Survivors	Nonsurvivors	*P* values
	(n = 102)	(n = 21)	(n = 81)	
**Source of patients, n (%)**				
Emergency room	50 (49.0)	12 (57.1)	38 (46.9)	0.403
Ward	48 (47.1)	5 (23.8)	43 (53.1)	0.017
Operating room	4 (3.9)	4 (19.0)	0 (0)	0.001
**Reasons for ICU admission, n (%)**				
Acute exacerbation of IIM alone	28 (27.5)	6 (28.6)	22 (27.2)	0.897
Infection alone	40 (39.2)	8 (38.1)	32 (39.5)	0.906
Concomitant infection and exacerbation of IIM	28 (27.5)	2 (9.5)	26 (32.1)	0.039
Others	6 (5.8)	5 (23.8)	1 (1.2)	0.001
**Main organ system affected at ICU admission, n (%)**				
Respiratory	78 (76.5)	13 (61.9)	65 (80.2)	0.206
Gastrointestinal	1 (1)	0 (0)	1 (1.2)	1.0
Neurologic	2 (2)	0 (0)	2 (2.5)	1.0
Cardiovascular	13 (12.7)	2 (9.5)	11 (13.1)	0.897
Renal	1 (1)	1 (4.8)	0 (0)	1.0
Others	7 (6.9)	6 (28.6)	1 (1.2)	< 0.001
**Laboratory findings at ICU admission**				
PaCO_2_, mean ± SD	39.2±9.8	37.3±9.6	39.7±9.8	0.314
PaO_2_/FiO_2_ ratio, median (IQR)	98.6 (68.0, 150.0)	225.0 (121.0,270.8)	85.0 (63.5,118.3)	<0.001
Creatine kinase, median (IQR)	54 (32,111)	43 (26,378)	55 (34,107)	0.886
ESR, median (IQR)	23 (13, 41)	16 (12,34)	37 (23,58)	0.230
hsCRP, median (IQR)	20.0 (1.9, 57.0)	10.7 (1.2, 51.9)	21.9 (2.4, 73.2)	0.216
Albumin (g/L), mean±SD	25±4	26±4	25±4	0.198
Creatinine (μmol/L), median(IQR)	70.0 (52.8, 85.5)	76.0 (51.0, 102.5)	69.5 (52.3, 85.0)	0.461
Total bilirubin (μmol/L), median (IQR)	11.0 (8.9,14.8)	11.6 (9.6, 14.5)	10.9 (8.4, 14.9)	0.585
WBC (x10^9^/L), median (IQR)	8.8 (7.0,13.2)	9.5 (6.5,18.1)	8.8 (7.0,12.6)	0.959
Lymphocyte count (per uL), median (IQR)	435 (227,693)	580 (435,1350)	360 (195,650)	0.013
Platelet (x10^12^/L), median (IQR)	129 (94,182)	120 (84,192)	132 (100,179)	0.516
APACHE II score, median (IQR)	17 (14,20)	13 (9, 19)	18 (15, 21)	0.010
SOFA score on ICU day1, median (IQR)	7 (4, 9)	4 (2,9)	8 (5, 9)	0.019
**Organ failure at ICU admission**				
Acute respiratory failure, n (%)	82 (80.4%)	8 (38.1%)	74 (91.4%)	< 0.001
Shock, n (%)	46 (45.1%)	5 (23.8%)	41(50.6%)	0.028
Acute kidney injury, n (%)	19 (18.6%)	3 (14.3%)	16 (19.8%)	0.796
**Interventions during ICU stay, n (%)**				
Noninvasive ventilation	16 (15.7)	4 (19.0)	12 (14.8)	0.980
Invasive mechanical ventilation	97 (95.1.)	17 (81.0)	80 (98.8)	0.005
Renal replacement therapy	10 (9.8)	1 (4.8)	9 (11.1)	0.645
Use of vasopressors	51(50.0)	9 (42.9)	42 (51.9)	0.463
**ICU-acquired infection**	11 (10.8)	1 (4.8)	10 (12.3)	0.546
**ICU length of stay (days), median (IQR)**	8.0 (5.0,13.3)	9.0 (1.0,13.5)	8.0 (5.0,13.5)	0.555
**Hospital length of stay (days), median (IQR)**	15.0 (9.0, 26.0)	29.0 (13.5, 47.0)	13.0 (8.0, 21.0)	<0.001

APACHE II, Acute Physiology and Chronic Health Evaluation II; ESR, erythrocyte sedimentation rate; hsCRP, high-sensitivity C-reactive protein; ICU, Intensive Care Unit; DM, dermatomyositis; PM, polymyositis; IQR, interquartile range; SOFA, sequential organ failure assessment.

At ICU admission, creatine kinase was elevated in 13 of 102 patients (12.7%). Forty-two patients (41.2%) had leukocytosis (white cell count >10,000/μL), while only 5 patients (4.9%) had leukocytopenia (white cell count <4,000/μL). Lymphocytopenia (lymphocyte count <1000/μL) was observed in 84 patients, and 54 patients had an absolute lymphocyte count <500/μL.

Ninety-seven (95.1%) patients received invasive mechanical ventilation, including 16 patients in whom an initial trial of noninvasive ventilation failed. Because of refractory hypoxemia, 26 patients received rescue therapies including the use of muscle relaxants, prone positioning, and high frequency oscillatory ventilation. Unfortunately, mortality among these patients reached 91.7%. In addition, a total of 51 patients (50%) were treated with vasopressors and 10 patients required continuous renal replacement therapy because of AKI. Furthermore, 18 patients received methylprednisolone pulse therapy and 25 patients received IVIG during their ICU stay.

### Patient outcomes and risk factors

The lengths of ICU/hospital stay were 8.0 days (5.0, 13.0) and 15.0 days (9.0, 26.0) respectively. Eighty-one patients died in the ICU, and 1 patient died in the general ward after ICU discharge, corresponding to an ICU/hospital mortality of 79.4% and 80.1% respectively. Causes of death in the ICU included refractory respiratory failure (n = 59, 72.8%), multiple organ failure (n = 13, 16.0%), refractory circulatory shock (n = 8, 9.8%), and cerebral hernia (n = 1, 1.2%).

Compared with survivors in the ICU, nonsurvivors were more likely to be male, diagnosed with DM/CADM, complicated with ILD, and to have been treated with methylprednisolone pulse. More nonsurvivors had been transferred from the general ward and admitted to the ICU because of the coexistence of infection and IIM exacerbation. On ICU admission, nonsurviors had had lower PaO_2_/FiO_2_ ratios, lower peripheral blood lymphocyte counts, higher severity-of-illness scores (APACHE II score and SOFA score); their condition had more frequently been complicated by acute organ failure (acute respiratory failure, shock). More nonsurvivors had also received invasive ventilation during their ICU stay (Tables 1 and 3).

**Fig 1:**
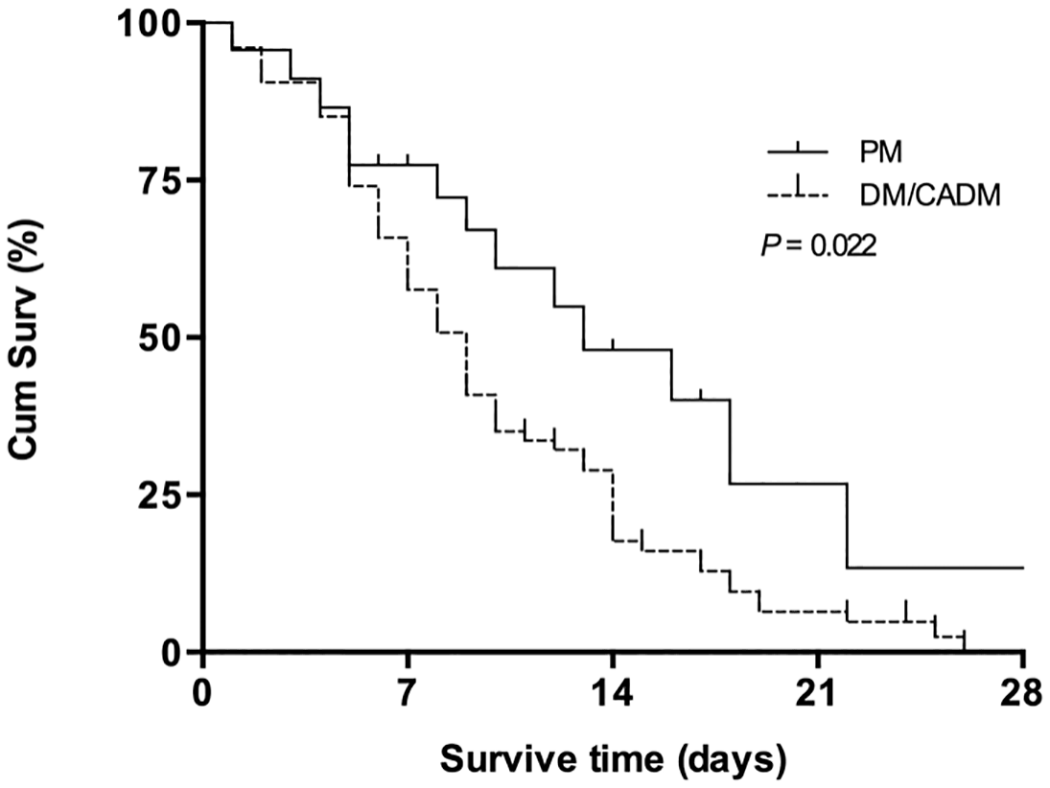
Kaplan-Meier analysis of survival probabilities in patients in ICU with PM and with DM/CADM. In the ICU, patients with PM had a significantly higher survival rates than those with DM/CADM. Survival time was censored on ICU day 28. The 2 groups were compared using the log-rank test (P = 0.022). CADM, Clinically Amyopathic Dermatomyositis; Cum Surv, cumulative survival; DM, dermatomyositis; ICU, Intensive Care Unit; PM, polymyositis.

Multivariate logistic regression analysis showed that the diagnosis of DM/CADM, the higher APACHE II score, and the presence of ARF at ICU admission and significantly increased the likelihood of failure to survive in the ICU. With regard to laboratory parameters at ICU admission, low peripheral blood lymphocyte counts and PaO_2_/FiO_2_ ratios were independent risk factors for ICU mortality (Table 4). The Kaplan-Meier survival curves showed that patients with DM/CADM had worse outcomes than patients with PM (*P* = 0.022) (Fig 1).

**Table 4:** Risk factors of mortality in the ICU: results of multivariate logistic regression analysis.

	Model 1^a^		Model 2^b^	
Variables	OR (95% CI)	*P* value	OR (95% CI)	*P* value
Diagnosis of DM/CADM	13.52(2.42, 75.57)	0.003	18.11 (3.53, 93.00)	0.001
APACHE II score	1.18 (1.01, 1.37)	0.036		
ARF at ICU admission	6.51 (1.61, 26.29)	0.008		
Lymphocytes at ICU admission (decrease per 100/μL)			1.30 (1.08, 1.56)	0.005
PaO_2_/FiO_2_ ratio at ICU admission (decrease per 50 mmHg)			1.36 (1.01, 1.8)	0.045

^a^ Variables in model 1 included gender, subtype of DM/CADM, ILD, respiratory muscle involvement, methylprednisolone pulse therapy within 1 month before or after ICU admission, admission from a general ward, concomitant infection and exacerbation at ICU admission, APACHE II score and SOFA scores (day1), presence of acute respiratory failure at ICU admission, septic shock at ICU admission, and mechanical ventilation. Goodness of fit for this model: chi-square *P* value 0.068.

^b^ Variables in model 2 included gender, subtype of DM/CADM, ILD, respiratory muscle involvement, methylprednisolone pulse therapy within 1 month before or after ICU admission, admission from general ward, concomitant infection and exacerbation at ICU admission, APACHE II score, lymphocyte absolute value at ICU admission, PaO_2_/FiO_2_ ratio at ICU admission. Goodness of fit for this model: chi-square *P* value 0.126.

OR, odds ratio; CI, confidence interval; ARF, acute respiratory failure; APACHE II, acute physiology and chronic health evaluation II; DM, dermatomyositis; ICU, intensive care unit.

## Discussion

IIM patients can have complicated life-threatening conditions requiring ICU care, posing a major clinical challenge for intensivists. Studies focusing on the outcomes and prognostic factors for this group of patients were scarce and were mostly embedded in a group of miscellaneous SRDs [5, 12, 22]. In the present study we retrospectively investigated the clinical features and outcomes of 102 ICU-admitted patients exclusively with IIMs. Thus, to the best of our knowledge, we were studying the first large cohort of such patients to date. We found that the outcomes for IIM patients admitted to the ICU were extremely poor, with a mortality of 79.4%, while the APACHE II score, presence of ARF, PaO_2_/FiO_2_ ratio, lymphocyte count at ICU admission, and a diagnosis of DM (including CADM) were independent prognostic factors for ICU mortality.

Previous studies have shown that the mortality of SRD patients in ICU as a whole was higher than that of non-SRD patients by 2- to 5-fold [23–25]. However, in the literature, the ICU mortality of patients with SRDs varied from 11% to 66.6%, depending on the type of underlying diseases [26–28]. Limited data showed that patients with IIM in the ICU had worse outcomes than those with other SRDs [3, 14]. Faguer’s study pointed to an 8-fold increase in odds of death among patients with DM [3]. The ICU mortality of IIM patients in the present study was comparable to that reported in Lee’s study (67%) [14] but much higher than that reported in Sherer’s study (33%) [15]. This discrepancy may be explained in part by severity of illness, organ involvement, or racial differences among populations. For instance, compared with those in Sherer’s study, the patients in the current study were older (50 years vs. 38 years); more patients also had an ILD (74.5% vs. 33.3%); and the latter patients had received high doses of steroids before ICU admission (69.7% vs. 50%). Selective referral bias might be another explanation. The patients seen at a large tertiary referral center, just like our hospital, represented a population with more severe or refractory disease and thus had less favorable outcomes.

A number of studies had demonstrated that ARF was the most common organ dysfunction that warranting intensive care in almost all SRD patients [5, 12], whereas there were no antecedent evidence suggesting that ARF was a predictor of mortality. However, our study consistently revealed that ARF and a decreased PaO_2_/FiO_2_ ratio at ICU admission were independent risk factors for ICU mortality in two separated multivariate regression models, which indicated that acute deterioration of respiratory function played a crucial role in the poor prognosis of IIM patients in the ICU. Similarly, in a retrospective study of 66 SRDs patients admitted to the ICU because of ARF, J. Lee and colleagues found that ICU-mortality in the IIM patients was relatively high among all those with SRDs [14]. The difference in the prognostic value of ARF between IIM and other SRDs may be due to the diversity of causes for ARF. In patients with systemic lupus erythematosus and systemic vasculitis, bacterial pneumonia and diffuse alveolar haemorrhage were the common causes for ARF, and these patients tended to respond well to treatment [5, 24]. On the other hand, in patients with IIM, the causes for ARF were mainly RP-ILD and opportunistic lung infections superimposed on ILD, both of which were often refractory to treatments [29–31]. Moreover, ARF in our cohort of patients was more severe owing to the rigorous definition of ARF that we used (PaO_2_/FiO_2_ <200 with mechanical ventilation); thus these patients had a higher risk of death.

The present study suggests that, after adjusted for the covariates, patients with DM/CADM had a higher risk of ICU death than those with PM. An association of specific IIMs with outcomes was also observed in the subgroup of IIM patients with ILD [29, 32]. The reasons for this different in prognosis between patients with DM/CADM and those with PM have not been elucidated; presumably they are related to the pathogenic mechanisms and reaction to immunosuppressive treatments. For example, histological study revealed a higher proportion of diffuse alveolar damage in the lungs of DM/CADM patients than in those with PM [29]. In addition, patients with DM/CADM were more refractory to immunosuppressive therapy than those with PM [32].

We found that lymphocytopenia was an independent risk factor for ICU mortality, as a decrease in peripheral blood lymphocyte of 100/μL was associated with a 30% increase in the likelihood of ICU death. A high prevalence of lymphocytopenia has been reported in untreated patients with IIM, and this alteration was correlated with disease activity [33, 34]. Viguier and coworkers, in a prospective study involving 47 patients with DM, found that before the initiation of therapy, lymphocytopenia (<1000/μL) occurred in 29 patients [33]. Moreover, the use of steroids and other immunosuppressants for the treatment of IIM inevitably increased the risk of reducing the lymphocyte count in peripheral blood. In the present study, almost 90% of patients were treated with high doses of corticosteroids or immunosuppressants and 25% received methylprednisolone pulse therapy 1 month prior to ICU admission. The association of lymphocytopenia with severe infection, especially opportunistic infection, has been well documented in patients with SRDs [9, 31, 33, 35]. Furthermore, it is well established that opportunistic infections are a frequent cause of death in patients with SRDs [36, 37]. A systematic review of infection-related mortality in SRD patients demonstrated that the mortality of PM/DM patients with *P*. *jiroveci* pneumonia was as high as 56% [37]. Hellmann and associates, in an analysis of the fatal infections in 44 patients with systemic lupus erythematosus patients, found that 77% of the death due to infection in this group were caused by opportunistic pathogens [36]. Our findings were in accordance with these results, as we observed that 29% of the patients in our study were admitted to ICU because of opportunistic infections and that the mortality among these patients was 83.3%.

With regard to opportunistic infection, the lung was the predominant infectious site; *Aspergillus* and *P*. *jiroveci* were the major pathogens in our study. In contrast, in a study of 104 hospitalized DM/PM patients with infection, Marie and colleagues reported that esophageal candidiasis and mycobacterial infection were the major opportunistic infections [9]. These differences may be due to different patient populations (ICU patients vs. patients in general wards). In the setting of the ICU, lower respiratory tract specimens are easier to obtain from intubated patients, and such specimens have higher yields of *Aspergillus* and *P*. *jiroveci*. Overall, the patients with IIM admitted to the ICU are at high risk for developing opportunistic pulmonary infections; in them aggressive immunosuppressive therapy should be implemented with caution.

The strengths of this study were that (1) the large sample of this IIM cohort made it possible achieve substantial conclusions and avoid intrinsic heterogeneity among different SRDs and (2) we took into account the coexistence of infection and acute exacerbation of underlying IIM as a distinct cause category of ICU admission. This is a common clinical scenario but was had not been fully considered in previous studies [24, 38, 39]. We acknowledge that it is sometimes difficult to identify causative factors accurately and that such judgments might be arbitrary. To overcome this, a case-by-case discussion was conducted by rheumatologists and intensivists. This study also has limitations to consider: our data were collected in a single center and might not represent the overall IIM care nationwide. Moreover, the quality of our data may have been suboptimal because of the retrospective design. However, given the rarity of IIMs in ICU patients, a prospective study would have been difficult to conduct.

## Conclusions

In summary, our findings highlighted the extremely poor prognoses of patients with IIM in ICU. APACHEII score, the presence of ARF, PaO_2_/FiO_2_ ratio, lymphocyte counts at ICU admission, and the diagnosis of DM (including CADM) were independent risk factors for ICU mortality. The complex etiology of the ARF, involving opportunistic infections and RP-ILD, distinguished IIM as a distinct entity as compared with other SRDs.

## Supporting Information

S1 File STROBE checklist. (DOC)

S1 Table Primary data of 102 patients with idiopathic inflammatory myopathy admitted to ICU. (XLSX)
